# Peripheral immune indicators and their predictive value in disease progression or relapse of pediatric Langerhans cell histiocytosis

**DOI:** 10.1016/j.jped.2025.101466

**Published:** 2025-11-02

**Authors:** Hua-Lin Li, Hong-Yun Lian, Wen-Yu Gong, Shuo Tian, Wei-Jing Li, Qing Zhang, Chan-Juan Wang, Hong-Hao Ma, Dong Wang, Yun-Ze Zhao, Zi-Jing Zhao, Jia-Jia Dong, Zhi-Gang Li, Rui Zhang, Lei Cui

**Affiliations:** a National Center for Children’s Health, Capital Medical University, Beijing Children's Hospital, Beijing Pediatric Research Institute, Laboratory of Hematologic Diseases, Beijing, China; b Capital Medical University, National Key Discipline of Pediatrics, Beijing, China; c Ministry of Education, Key Laboratory of Major Diseases in Children, Beijing, China; d National Center for Children’s Health, Capital Medical University, Beijing Children’s Hospital, Department of Hematology, Beijing, China

**Keywords:** Lymphocyte subsets, Cytokines, Pediatric langerhans cell histiocytosis, Progression-free survival

## Abstract

**Objective:**

Langerhans cell histiocytosis (LCH) is a rare inflammatory myeloid neoplasm in which the inflammatory microenvironment plays a crucial role in the development and progression of disease. The prognostic value of circulating lymphocyte subsets and cytokines remains uncertain.

**Methods:**

The authors retrospectively analyzed baseline peripheral lymphocyte subsets and serum cytokines in 330 consecutive pediatric patients. Immune profiles were compared across disease extent, clinical events, and biological features. Prognostic associations with progression-free survival (PFS) were tested using univariable and multivariable models.

**Results:**

Peripheral immune profiles varied with disease extent. Patients with multisystem risk-organ involvement (MS RO^+^) had fewer total T and Th1 cells, more CD4⁺ T and B cells, and higher IL-6, IL-10, and IFN-γ. Patients who progressed or relapsed showed a similar pattern, and non-survivors had particularly high IL-10. In the first-line cohort, the proportions of T, B, CD4⁺ T, CD8⁺ T, and Th1 cells, ratios of CD4/CD8 and Th1/Th2, and levels of IL-6, IL-10 predicted progression/relapse, and Youden-derived cut-offs dichotomized with distinct PFS. On multivariable Cox, IL-6, IL-10, Th1/Th2 ratio, RO status, and week-6 responses were independent predictors, and a nomogram model with good predictive capability was formed. IL-10 remained independently prognostic in multisystem LCH; the immune indices were not prognostic in single-system LCH. External validation in 103 patients confirmed risk stratification and model performance with well-calibrated PFS estimates.

**Conclusion:**

Baseline peripheral lymphocyte subsets and cytokines carried prognostic information in pediatric LCH. The IL-6, IL-10, and Th1/Th2 profile supported risk stratification and may inform treatment planning.

## Introduction

Langerhans cell histiocytosis (LCH), the most common histiocytic disorder in children, features lesions of aberrant CD1a/CD207 (langerin)–expressing Langerhans cells with immune infiltrates [1,2]. Disease course spans from self-limited unifocal involvement to life-threatening multisystem (MS) disease. Activating MAPK-pathway mutations–most commonly *BRAF*-V600E and *MAP2K1*–drive pathogenesis of LCH [3,4]. Despite improved outcomes with risk-adapted therapy, about one-third of patients relapsed [5,6]. MAPK inhibitors benefit refractory/recurrent LCH, but relapse is frequent after discontinuation, highlighting the need for reliable prognostic tools [7,8].

The inflammatory microenvironment is central to LCH pathogenesis. Lesions contain prominent myeloid and T-cell infiltrates that secrete exuberant pro-inflammatory cytokines and chemokines [9]. Lesional T cells frequently display dysfunction and exhaustion, with a relative excess of CD4^+^ over CD8^+^ *T* cells [10,11]. Regulatory T cells (Tregs) are enriched in lesions and increased in the circulation during active disease [12]. These cellular interactions and signaling networks support lesion persistence and dissemination [13].

Peripheral immune markers track with LCH disease extent and treatment response. Compared with single-system (SS) disease, MS involvement is associated with higher cytokine/chemokine levels–such as soluble interleukin-2 receptor (sIL-2R) and interleukin-18 (IL-18)–at baseline [14]. Risk-organ involvement (RO^+^) correlates with elevated IL-6, IL-10, interferon-gamma (IFN-γ), increased activated T cells, and poorer treatment responses [15]. sIL-2R and tumor necrosis factor-alpha (TNF-α) help identify MS RO^+^ patients at diagnosis, and sIL-2R and CD8^+^ *T* cells decline significantly after treatment [16]. Higher baseline sIL-2R levels were also associated with high-risk features and worse prognosis [17]. Nonetheless, the prognostic value of circulating lymphocyte subsets and cytokines in LCH remains incompletely elucidated. The authors therefore conducted a retrospective study to examine associations among peripheral lymphocyte subpopulations, cytokine levels, clinical characteristics, and outcomes in LCH patients, and to evaluate their utility for prognostic stratification.

## Methods

### Enrolled patients

From January 2017 to September 2020, 368 newly diagnosed pediatric LCH patients were identified through the electronic medical records of Beijing Children's Hospital [18]. Among them, 330 patients met the inclusion criteria: confirmed LCH diagnosis, age < 18 years, complete baseline peripheral lymphocyte subsets and cytokines testing, and standardized treatment with follow-up. Thirty-eight were excluded due to missing data or loss to follow-up (Supplementary Figure S1).

The Ethics Committee of Beijing Children’s Hospital approved this study. Written informed consent was exempted due to the retrospective design, with all personal identifiers securely protected.

### Sample collection

Peripheral blood for lymphocyte subset profiling and cytokine measurement was collected at diagnosis, before the start of any systemic therapy. The samples were immediately processed as fresh specimens. None of the patients had received systemic corticosteroids before the baseline blood draw. Upon admission, all patients underwent standardized tests for pathogens, and no evidence of concurrent infection was identified.

### Lymphocyte subsets analysis by flow cytometry

Cells (stimulated or unstimulated) were stained for surface antigens using fluorochrome-conjugated monoclonal antibodies, then fixed and permeabilized for intracellular staining. Isotype controls with irrelevant specificity were run in parallel. Samples were acquired on a BD FACSCanto II, with lymphocytes defined by low side scatter and bright CD45 expression. CD3^+^ *T*, CD19^+^ *B*, and CD16^+^/CD56^+^ natural killer (NK) cells were subsequently assessed from total lymphocytes. The percentages of CD4^+^ and CD8^+^ *T* cells were derived from the CD3^+^ population. T helper 1 (Th1, defined as CD3^+^ CD8^-^ IFN-γ^+^), Th2 (CD3^+^ CD8^-^ IL-4^+^) and Th17 (CD3^+^ CD8^-^ IL-17^+^) subsets were calculated from CD3^+^ CD8^-^ cells. Tregs (CD3^+^ CD4^+^ CD25^+^ FoxP3^+^) were derived from CD3^+^ CD4^+^ cells. Ratios (CD4/CD8, Th1/Th2, and Treg/Th17) were calculated. Antibodies used are listed in Supplementary Table S1, and the gating strategy is depicted in Supplementary Figure S2.

### Cytokine profiling

Serum cytokines (IL-2, IL-4, IL-6, IL-10, TNF-α, and IFN-γ) were quantified by cytometric bead array (CBA, BD Human Th1/Th2 Cytokine Kit II, 551,809). Standards were prepared by serial dilution. Capture beads were incubated with serum samples, standards, and kit controls for three hours at room temperature, washed twice, and acquired by flow cytometry (FC, BD FACSCanto II). Data were analyzed with FCAP Array v3.0. Potential outliers — values ± 3 standard deviations (SD) from group means—were re-analyzed on raw fluorescence histograms; exclusions were limited to biologically implausible signals consistent with technical artifact (*e.g.*, instrument noise). Dynamic range was supported by antibody-titration optimization and per-run internal controls. Protocols followed peer-reviewed standards.

### Treatment protocol

Patients were stratified by disease extent: SS, MS RO^-^, and MS RO^+^ LCH. RO refers to the liver, spleen, or hematologic system. Treatment followed the BCH-LCH 2014 protocol [6]. First-line therapy consisted of six or twelve weeks of induction with vindesine and prednisone, followed by maintenance that included vindesine and prednisone, with or without 6-mercaptopurine. Second-line chemotherapy consisted of an intensified regimen of cytarabine, vindesine, and dexamethasone, with or without cladribine. Maintenance treatment then continued with vindesine, prednisone, and 6-mercaptopurine. Response was defined as non-active disease or better [19]. Disease progression was defined as clinical worsening or new lesions while on therapy. Relapse was defined as recurrence of active disease after complete remission or more than three months post-maintenance [20].

### Statistical analysis

Categorical variables were compared using Fisher's exact test, while continuous variables were analyzed with the Mann-Whitney U or Kruskal-Wallis test. Associations were assessed by Spearman correlation. Overall survival (OS) was defined as the time interval from diagnosis to death, and progression-free survival (PFS) was defined as the time from treatment initiation to disease progression, relapse, or death, whichever occurred first. The performance of binary predictions was evaluated using receiver operating characteristic (ROC) curves and the area under the curve (AUC); optimal cut-offs were determined by Youden's index. Variables with ROC *P* < 0.05 were included in the univariate analyses (Kaplan-Meier with log-rank test). Those with *P* < 0.10 progressed to the multivariable Cox model to identify independent prognostic factors. A prognostic nomogram was built with the “rms” R package (v6.3–0). The model’s ability to discriminate between outcomes was assessed using the concordance index (C-index). The calibration was evaluated through bootstrap-corrected calibration curves, utilizing 200 resamples. A composite risk score was calculated from the independent prognostic factors. Continuous indicators, specifically IL-6, IL-10, and the Th1/Th2 ratio, were processed via the “survival” R package (v3.3.1). Patients were categorized into high- and low-risk groups based on the median risk score. Analysis was performed in SPSS 25.0 (IBM) and R (v4.2.1); Visualizations, including donut plots, bubble heatmaps, correlation pie charts, and risk score plots, were generated with the ggplot2 package (v3.4.4). Unless otherwise specified, tests were two-sided, and *P* < 0.05 was considered statistically significant.

## Results

### Baseline characteristics and long-term outcomes

Baseline demographics of 330 enrolled patients are presented in Supplementary Figure S3A. The median diagnostic age was 2.8 years (0.5–16.1). Disease extent distribution included 158 (47.9 %) SS-LCH, 115 (34.8 %) MS RO^-^, and 57 (17.3 %) MS RO^+^-LCH. Involved organs included bone, skin, lung, liver, pituitary, lymph nodes, spleen, hematologic system, ear, eye, and, rarely, the thymus. Of 233 genotyped, *BRAF*-V600E was detected in 136 (58.4 %), *MAP2K1* variants in 21 (9.0 %), and other *BRAF* alterations in 17 (7.3 %). Baseline features did not differ between enrolled and excluded cohorts (Supplementary Table S2).

Initial management comprised first-line therapy (*n* = 294), upfront second-line chemotherapy for pituitary involvement (*n* = 10), targeted therapy with dabrafenib/trametinib for age or vascular constraints (*n* = 10), and observation for non-central nervous system (CNS)-risk single-site lesion (*n* = 16). Over a median follow-up of 73.0 months, three (0.9 %) deaths occurred (5-year OS, 99.1 % ± 0.5 %). Fifty-three patients (16.1 %) progressed and 97 (29.4 %) relapsed, yielding a 5-year PFS of 54.7 % ± 2.7 %. Permanent consequences (PCs) developed in 16 patients (five neurodegeneration, four liver cirrhosis, four anterior pituitary hormone deficiency, three diabetes insipidus) with a 5-year cumulative incidence of 3.1 % ± 1.0 % (Supplementary Figure S3B). Seventeen patients had diabetes insipidus at baseline.

### Comparison of lymphocyte subsets/cytokines by disease extent and events

**Figure 1:**
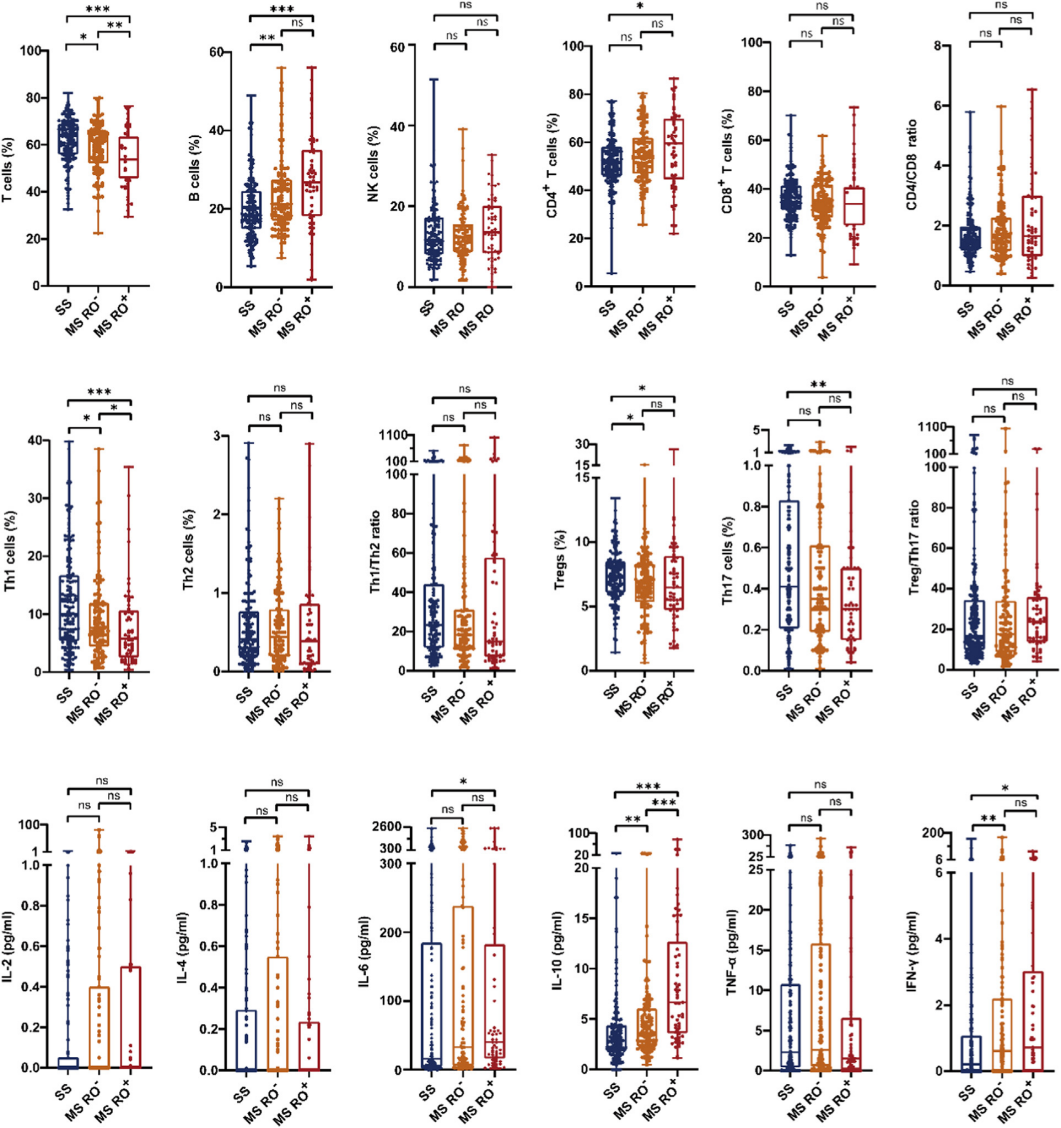
Box-and-whisker plots showing lymphocyte subset proportions and cytokine levels across the three clinical subtypes of LCH. Abbreviations: LCH, Langerhans cell histiocytosis; RO, risk organ; SS, single-system; MS, multisystem.

Across LCH subtypes (Figure 1), the percentages of T cells were lowest in MS RO^+^-LCH (*vs.* SS-LCH, *P* < 0.001; *vs.* MS RO^-^-LCH, *P* = 0.018, respectively), whereas B cells were lowest in SS-LCH (*vs.* MS RO^-^-LCH, *P* = 0.002; *vs.* MS RO^+^-LCH, *P* < 0.001, respectively). MS RO^+^ LCH showed higher CD4^+^ *T* cells (*P* = 0.014) but lower Th1, Tregs, and Th17 cells than SS-LCH (*P* < 0.001, *P* = 0.045, and *P* *=* 0.004, respectively), and higher IL-6, IL-10, and IFN-γ (*P* = 0.034, *P* < 0.001, and *P* = 0.010, respectively).

**Figure 2:**
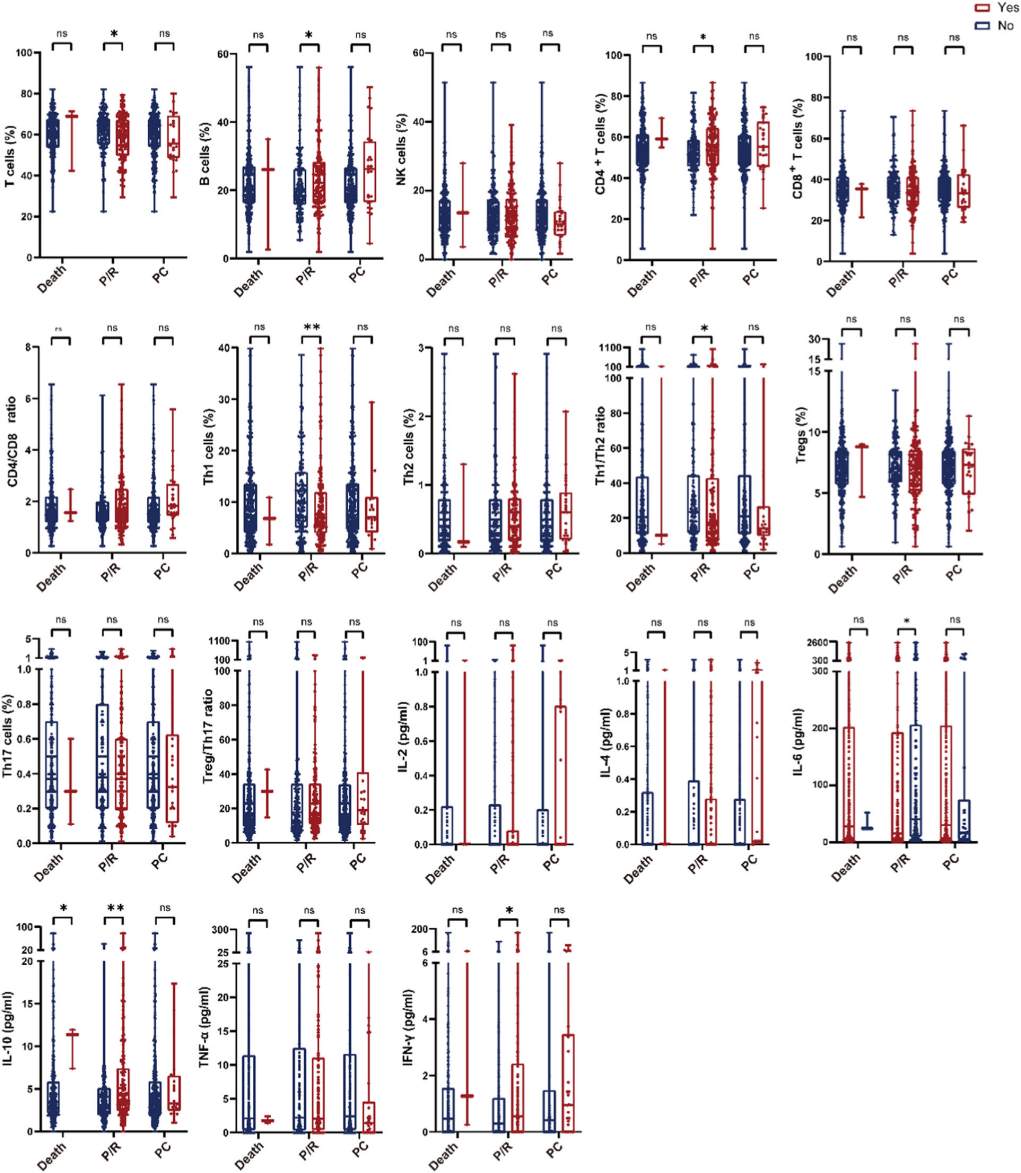
Box-and-whisker plots comparing lymphocyte subset proportions and cytokine levels by event status. Events included death, progression/relapse (P/R), and permanent consequences (PC).

Patients with progression/relapse had higher B and CD4^+^ *T* cells (*P* = 0.048, and *P* = 0.014, respectively) but lower total T cells, Th1 cells, and Th1/Th2 ratios (*P* = 0.033, 0.003, and 0.048, respectively), with elevated IL-6, IL-10, and IFN-γ (*P* = 0.013, 0.006, and 0.036, respectively); IL-10 was particularly higher in non-survivors (*P* = 0.020). PCs showed no association with lymphocyte subsets/cytokines (Figure 2). Within MS-LCH, lower total T and Th1 cell proportions and elevated IL-10 levels were associated with progression, relapse, or death (Supplementary Figure S4). No significant difference was observed in SS-LCH (Supplementary Figure S5).

### Correlation with clinical-biological features

At baseline (Supplementary Figure S6), age < 2 years associated with lower total T, CD8^+^ *T*, Th1, Th2, Th17, Tregs, and Th1/Th2 ratios, but higher B, CD4^+^ *T* cells, CD4/CD8 and Treg/Th17 ratios. *BRAF*-V600E carriers had fewer T cells and more B cells. By organ, skin involvement was associated with lower total T and Th1 cells and higher total B and CD4^+^ *T* cells; splenic or hematologic involvement correlated with lower Th17 cells and higher Treg/Th17 rations; pulmonary involvement was associated with lower Tregs. IL-6 and IL-10 levels were higher in younger age, *BRAF*-V600E positivity, or cutaneous disease; IFN-γ was higher with splenic or hematologic involvement (all *P* < 0.05).

Patients presenting with fever at diagnosis had higher IL-10 and IFN-γ at baseline compared to those without fever (*P* < 0.001 and *P* = 0.014, respectively). Among 20 patients with secondary Hemophagocytic lymphohistiocytosis (sHLH), levels of IL-10 and TNF-α were significantly elevated compared to other MS cases without sHLH (*P* < 0.001 and *P* *=* 0.025, respectively; Supplementary Figure S7). Additionally, Inter-marker correlations were generally weak, with most correlation coefficients below 0.2 (Supplementary Figure S8).

Age, sex, and mutation-related trends remained consistent when the cohort was divided into MS-LCH and SS-LCH (Supplementary Figure S9). The organ-specific patterns observed in MS-LCH were similar to those found in the overall cohort. In SS-LCH, immune profiles differed by site of involvement. Specifically, compared with cutaneous disease, bone involvement displayed higher proportions of Th1, CD8^+^ *T*, and NK cells and higher Th1/Th2 ratios, but lower proportions of CD4^+^ *T* and B cells and lower CD4/CD8 ratios (all *P* < 0.05).

### Prognostic value of immune indices

**Figure 3:**
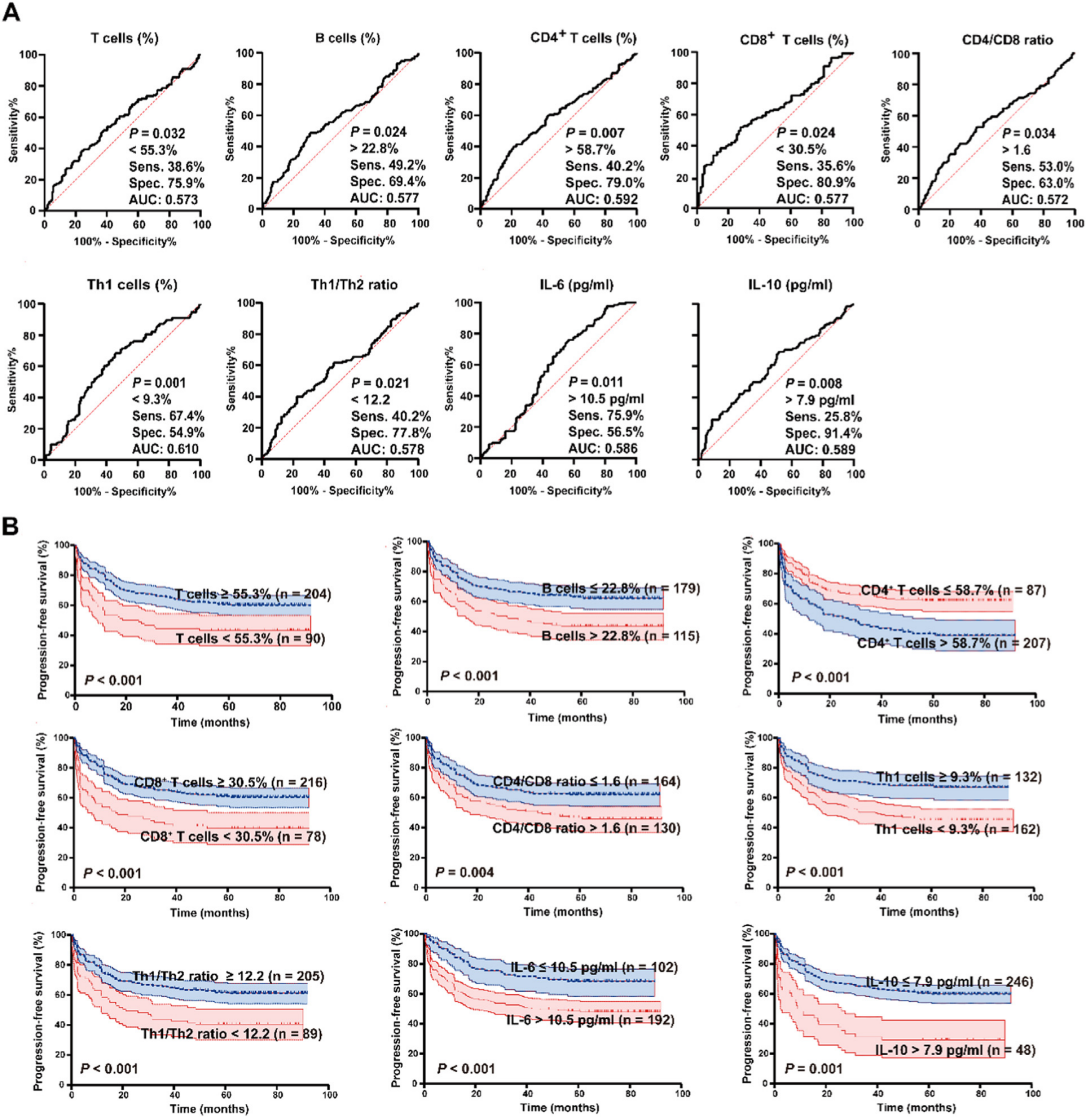
Prognostic significance of lymphocyte subsets and cytokines in pediatric Langerhans cell histiocytosis. (A) ROC curves for variables with significant predictive value (*P* < 0.05); full results are provided in Supplementary Table S3. (B) Kaplan-Meier survival curves comparing groups stratified by the cut-off values for lymphocyte subsets and cytokines, determined by Youden's index from the ROC analysis.

**Figure 4:**
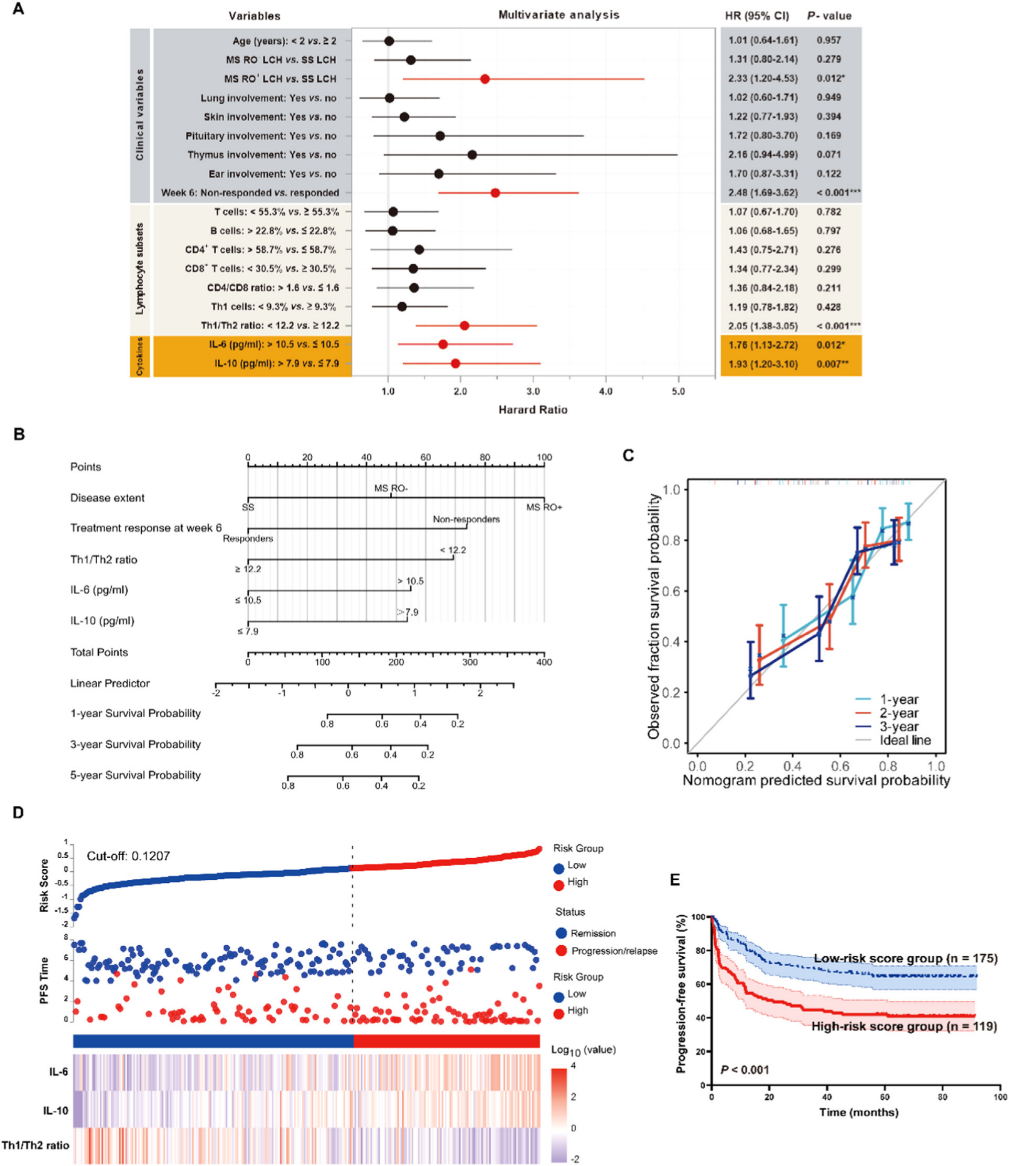
Multivariate analysis of risk factors for progression-free survival (PFS) in patients receiving first-line therapy. (A) Forest plot of the multivariate Cox regression. (B) Nomogram for PFS prediction. (C) Calibration curves at 1, 3, and 5 years. (D) Risk-score distribution, survival status, and heatmaps of IL-6, IL-10, and the Th1/Th2 ratio. (E) Kaplan-Meier survival curves for high- *vs*. low-risk score groups.

In 294 patients receiving first-line chemotherapy, the proportions of T, B, CD4^+^ *T*, CD8^+^ *T*, and Th1 cells, ratios of CD4/CD8 and Th1/Th2, and levels of IL-6, IL-10 predicted progression/relapse (ROC, Figure 3A, Supplementary Table S3). Youden-derived cut-offs dichotomized with distinct PFS (Figure 3B). The Prognostic significance of clinico-biological characteristics, lymphocyte subsets, and cytokines was assessed by univariate analysis (Supplementary Table S4). On multivariable Cox, IL-6, IL-10, Th1/Th2 ratio, RO status, and week-6 treatment response were independent prognostic factors (Figure 4A), informing a nomogram model with good predictive capability (C-index 0.715, 95 % CI 0.692–0.738, Figure 4B). Furthermore, the calibration plots indicated a good agreement between the predicted PFS rates and the actual observations (Figure 4C).

Additionally, the authors performed a risk score analysis to evaluate the predictive value of IL-6, IL-10, and Th1/Th2 ratio, treating them as continuous variables (Figure 4D). The authors then classified the patients into high-risk and low-risk score groups using the median risk score as a cut-off. The survival analysis revealed inferior PFS in the high-risk group compared to the low-risk group (*P* < 0.001; Figure 4E).

Across the three clinical subtypes of LCH, most of the three indicators (IL-6, IL-10, and Th1/Th2 ratio) showed significant or trending PFS differences (Supplementary Figure S10). Higher IL-6 tended to be associated with progression/relapse after treatment with BRAF/MEK inhibitors (*P* = 0.068). However, the authors did not observe significant differences among other treatment groups (Supplementary Figure S11). In MS-LCH, total T cells, Th1 cells, and IL-10 levels retained prognostic value by ROC analysis, and IL-10 remained independently prognostic in multivariable Cox models (Supplementary Table S5-S6 and Figure S12). In contrast, these immune indicators showed no significant prognostic relevance in SS-LCH (Supplementary Table S7).

### External validation

An independent validation cohort of 103 pediatric LCH patients diagnosed between October 2020 and June 2022 was included. There were 60 boys (58.3 %) and 43 girls (41.7 %), with a median age of 4.6 years (range 1.9–7.7). Disease subtypes included SS-LCH (50 cases, 48.5 %), MS RO^-^-LCH (34 cases, 33.0 %), and MS RO^+^-LCH (19 cases, 18.4 %). With a median follow-up of 45.0 months, the 3-year PFS rate was 61.2 % ± 4.8 %. Using the discovery cut-offs for Th1/Th2, IL-6, and IL-10, PFS differed significantly between groups (Supplementary Figure S13A). The prognostic nomogram model showed good discrimination in the validation cohort, achieving a C-index of 0.800 (95 % CI: 0.767–0.833) (Supplementary Figure S13B). Calibration plots showed strong agreement between predicted and observed PFS (Supplementary Figure S13C).

## Discussion

LCH exhibits features of both a neoplasia and an inflammatory disorder, and progression or recurrence remains a challenge. In a large cohort of 330 children with LCH, the authors profiled baseline lymphocyte subsets and cytokines and found consistent associations with disease extent and outcomes. Using independent immune risk factors, the authors then developed and validated a prognostic model to support risk stratification and management.

Prognosis in LCH varies by clinical category, with markedly higher mortality and relapse in patients with RO involvement [21]. Consistent with this, children with MS RO^+^ disease showed lower circulating total T and Th1 cells, higher CD4^+^ *T* and B cells, and elevated IL-6, IL-10, and IFN-γ. This RO^+^ immune profile mirrored that of patients who progressed or relapsed, indicating systemic inflammation and immune dysregulation consistent with LCH as an inflammatory myeloid neoplasm and associated with adverse outcomes [22]. The authors also observed that *BRAF*-V600E was associated with fewer T cells and expanded B cell compartments, consistent with the “misguided myeloid differentiation” model in which MAPK activation in hematopoietic precursors skews myeloid differentiation and promotes inflammatory cytokine production [23]. *BRAF* mutation was associated with increased IL-6 and IL-10, suggesting mutation-associated systemic inflammation and a tendency toward poorer outcomes. While BRAF/MEK inhibitors elicit rapid responses, relapse after discontinuation argues for combinatorial approaches targeting clonal precursors to secure durable remission [24,25].

Mechanistically, the present data suggested that the Th1/Th2 axis and the cytokines IL-6 and IL-10 were key factors correlated with risk. Th1 cells drive cell-mediated immunity through IFN-γ, IL-2, and TNF-α, activating macrophages and promoting differentiation of CD8^+^ *T* cells to enhance cytotoxic function. By contrast, Th2 cells support humoral responses via IL-4, IL-10, and IL-13, suppressing Th1-mediated responses and fostering an immunosuppressive tumor microenvironment [26]. In LCH, a reduced Th1/Th2 ratio signals Th2 skewing that may suppress cytotoxic surveillance and recruit pro-tumor myeloid populations, including myeloid-derived suppressor cells and M2 macrophages [27]. As a pro-inflammatory cytokine, IL-6 may sustain LCH-cell proliferation and survival by activating STAT3/MAPK signaling and promoting VEGF-dependent angiogenesis, contributing to lesion expansion and dissemination [28]. IL-10, produced by Th2 cells, Tregs, and M2 macrophages within lesions, attenuates immune surveillance by restraining dendritic-cell maturation and T-cell activation, facilitating immune escape [29]. Clinically, elevation of this triad (low Th1/Th2, high IL-6 and IL-10) was associated with more aggressive disease and systemic immunosuppression. Therapeutic normalization likely reflects remission and re-established immune homeostasis, whereas persistent dysregulation denotes ongoing dysfunction and elevated relapse risk. Demonstrating concordance between circulating markers and lesional immunity will require paired longitudinal cohorts.

The prognostic nomogram and risk-score integrating IL-6, IL-10, and Th1/Th2 provided incremental prognostic value beyond clinical subtype and genotype, particularly for identifying children at risk of early progression or recurrence. A trend toward higher IL-6 among patients relapsing on targeted therapy further supports exploring IL-6 as a dynamic monitoring biomarker to guide response-adaptive interventions. Limitations include the absence of longitudinal monitoring of these indicators during treatment, which should be addressed in future prospective designs. Besides, cytokines were quantified by FC, which has a narrower dynamic range compared to immunoassays like ELISA. While the authors optimized the assays and focused on relative differences, complementary platforms may allow for absolute quantification across a broad range of concentrations. Additionally, fever and sHLH may confound cytokine measurements. The authors pre-specified immune predictors and used a two-stage screening procedure to limit multiplicity and overfitting, yet evaluating correlated biomarkers still carried risk. In an independent external cohort, the model showed good calibration and a C-index of 0.800, supporting its generalizability. Even so, larger prospective studies with longitudinal immune profiling are needed, and future work should examine integration with other biomarkers (*e.g.*, C-reactive protein) and additional immune readouts.

Stratified analyses indicated that immune profiles differed by disease type and were prognostically informative mainly in MS-LCH. In MS-LCH, lower total T and Th1 cells with higher IL-10 were associated with progression/relapse, and IL-10 remained independently predictive of PFS after adjustment. These markers were not prognostic in SS-LCH, suggesting that local lesion biology may dominate risk in single-system disease. The absence of prognostic signal in SS-LCH may also reflect fewer progression/relapse events, limiting statistical power. Clinically, the present data support disease–type–specific risk stratification—incorporating IL-10 for MS-LCH—while recognizing limited utility in SS-LCH. Prospective, longitudinal validation should assess whether dynamic changes in these indices can guide response-adaptive therapy.

In summary, circulating lymphocyte subsets and cytokines carried prognostic information in pediatric LCH and supported disease type-specific risk stratification. Given that LCH lies at the intersection of clonal oncogenic signaling and immune dysregulation, optimal management should integrate molecular mutation status with immune-microenvironment features. Deeper, longitudinal profiling of cytokines and T-cell subsets—particularly IL-10, IL-6, and Th1/Th2 balance — may refine risk assessment and inform timing and selection of targeted and immunomodulatory therapies.

## Authors' contributions

Hua-Lin Li and Hong-Yun Lian analyzed the data and wrote the manuscript. Wen-Yu Gong and Shuo Tian performed the experiments. Wei-Jing Li, Qing Zhang, Chan-Juan Wang collected the clinical information. Hong-Hao Ma, Dong Wang and Yun-Ze Zhao made clinical contributions. Zi-Jing Zhao and Jia-Jia Dong collected the samples. Zhi-Gang Li contributed to data analysis. Lei Cui and Rui Zhang designed the research study and revised the paper. All authors were involved in the final approval of the paper.

## Funding

This work was supported by the Beijing Natural Science Foundation (No. 7242053 and 7254350), Capital’s Funds for Health Improvement and Research (No. 2022–2–1141), and Funding for Reform and Development of Beijing Municipal Health Commission (EYGF-XY-06).

## Conflicts of interest

The authors declare no conflicts of interest.

## Data Availability

The de-identified dataset supporting the findings is available from the corresponding author upon reasonable request and subject to institutional approvals and a data use agreement. Analysis scripts will be shared with the request.

## Appendix Supplementary materials

Image, application 1

## Footnotes

Supplementary material associated with this article can be found in the online version at doi:10.1016/j.jped.2025.101466.
