# Supplementary material for: Peripheral immune indicators and their predictive value in disease progression or relapse of pediatric Langerhans cell histiocytosis
Source: J Pediatr (Rio J). 2025 Nov 2;101(6):101466. doi: 10.1016/j.jped.2025.101466 (PMC12621562; doi:10.1016/j.jped.2025.101466)
Supplement: Supplementary file 1 [file mmc1.docx]

**JPED-D-25-00274_Supplementary Material**

**Supplementary Table S1** The antibodies used to detect lymphocyte subsets by flow cytometry.

| Antigen | Fluorochrome | Clone | Catalog | Manufacturer |
| --- | --- | --- | --- | --- |
| CD3 | [PerCP](https://www.bdbiosciences.com/zh-cn/products/reagents/flow-cytometry-reagents/research-reagents/single-color-antibodies-ruo/percp-mouse-anti-human-cd3.552851) | SP34-2 | 347344 | Biolegend, USA |
| CD25 | [APC](https://www.bdbiosciences.com/en-us/products/reagents/flow-cytometry-reagents/clinical-discovery-research/single-color-antibodies-ruo-gmp/apc-r700-mouse-anti-human-cd25.659126) | 2A3 | 340938 | Biolegend, USA |
| CD8 | [APC](https://www.bdbiosciences.com/en-us/products/reagents/flow-cytometry-reagents/clinical-discovery-research/single-color-antibodies-ruo-gmp/apc-r700-mouse-anti-human-cd25.659126) | SK1 | 340659 | Biolegend, USA |
| CD4 | [FITC](https://www.bdbiosciences.com/en-us/products/reagents/flow-cytometry-reagents/clinical-discovery-research/single-color-antibodies-ruo-gmp/apc-r700-mouse-anti-human-cd25.659126) | SK3 | 340133 | Biolegend, USA |
| IFN-γ | [FITC](https://www.bdbiosciences.com/en-us/products/reagents/flow-cytometry-reagents/clinical-discovery-research/single-color-antibodies-ruo-gmp/apc-r700-mouse-anti-human-cd25.659126) | TgMab-2 | 340456 | Biolegend, USA |
| IL-4 | [PE](https://www.bdbiosciences.com/en-us/products/reagents/flow-cytometry-reagents/clinical-discovery-research/single-color-antibodies-ruo-gmp/apc-r700-mouse-anti-human-cd25.659126) | 3010.211 | 340456 | Biolegend, USA |
| FOXP3 | PE | PCH101 | 12-4776-42 | Invitrogen, USA |
| IL-17A | APC-Cy7 | PCH101 | 47-7179-42 | Invitrogen, USA |
| CD3 | FITC | UCHT1 | Z6410010 | Tongsheng Shidai, China |
| CD19 | APC | HIB19 | Z6410010 | Tongsheng Shidai, China |
| CD16 | PE | CB16 | Z6410010 | Tongsheng Shidai, China |
| CD56 | PE | MEM-188 | Z6410010 | Tongsheng Shidai, China |
| CD45 | PerCP-Cy5.5 | 2D1 | Z6410010 | Tongsheng Shidai, China |

**Supplementary Table S2** Comparison of clinical features in patients with Langerhans cell histiocytosis (LCH) included (n = 330) or excluded (n = 38) in this study.

| Variables | n (%) | Included | Excluded | *P*-value |
| --- | --- | --- | --- | --- |
| All | 368 | 330 | 38 |  |
| Gender |  |  |  |  |
| Male | 229 (62.2) | 208 (63.0) | 21 (55.3) | 0.350 |
| Female | 139 (37.8) | 122 (37.0) | 17 (44.7) |  |
| Age (years), median (range) | 2.7 (1.2-5.6) | 2.8 (1.3-5.6) | 2.5 (0.6-5.8) | 0.475 |
| ≥ 2 | 226 (61.4) | 205 (62.1) | 21 (55.3) | 0.411 |
| < 2 | 142 (38.6) | 125 (37.9) | 17 (44.7) |  |
| Disease extent |  |  |  |  |
| SS LCH | 175 (47.5) | 158 (47.9) | 17 (44.7) | 0.080 |
| MS RO^－^LCH | 124 (33.7) | 115 (34.8) | 9 (23.7) |  |
| MS RO^+^LCH | 69 (18.8) | 57 (17.3) | 12 (31.6) |  |

**Supplementary Table S3** Receiver operating characteristic (ROC) curve analysis of lymphocyte subsets and cytokines in predicting prognosis of children with Langerhans cell histiocytosis (LCH) treated with first-line therapy.

| Indicators | AUC | 95% CI | *P*-value | Cut-off value |
| --- | --- | --- | --- | --- |
| Lymphocyte subsets (%) |  |  |  |  |
| T cells | 0.573 | 0.506-0.639 | 0.032 | 55.3 |
| B cells | 0.577 | 0.510-0.643 | 0.024 | 22.8 |
| NK cells | 0.506 | 0.342-0.669 | 0.945 | 11.8 |
| CD4^+^ T cells | 0.592 | 0.526-0.658 | 0.007 | 58.7 |
| CD8^+^ T cells | 0.577 | 0.510-0.643 | 0.024 | 30.5 |
| CD4/CD8 ratio | 0.572 | 0.505-0.639 | 0.034 | 1.6 |
| Th1 cells | 0.610 | 0.544-0.675 | 0.001 | 9.3 |
| Th2 cells | 0.505 | 0.439-0.572 | 0.877 | 0.3 |
| Th1/Th2 ratio | 0.578 | 0.512-0.645 | 0.021 | 12.2 |
| Th17 cells | 0.522 | 0.455-0.589 | 0.522 | 5.2 |
| Tregs | 0.545 | 0.479-0.611 | 0.187 | 0.8 |
| Treg/Th17 ratio | 0.524 | 0.457-0.590 | 0.488 | 12.0 |
| Cytokines (pg/ml) |  |  |  |  |
| IL-2 | 0.507 | 0.440-0.574 | 0.837 | 0.1 |
| IL-4 | 0.545 | 0.479-0.611 | 0.184 | 1.0 |
| IL-6 | 0.586 | 0.522-0.651 | 0.011 | 10.5 |
| IL-10 | 0.589 | 0.523-0.655 | 0.008 | 7.9 |
| TNF-α | 0.505 | 0.438-0.571 | 0.891 | 1.3 |
| IFN-γ | 0.566 | 0.499-0.632 | 0.052 | 2.3 |

**Supplementary Table S4** Univariate analysis of risk factors for progression-free survival (PFS) of LCH patients.

| Variables | Groups | n (%) | 5-year PFS (% ± SE) | *P*-values |
| --- | --- | --- | --- | --- |
| Age | < 2 years | 107 (36.4) | 41.0 ± 4.8 | < 0.001 |
|  | ≥ 2 years | 187 (63.6) | 62.9 ± 3.6 |  |
| Disease extent | SS LCH | 142 (48.3) | 65.3 ± 4.0 | < 0.001 |
|  | MS RO^－^LCH | 105 (35.7) | 51.2 ± 4.9 |  |
|  | MS RO^+^ LCH | 47 (16.0) | 31.9 ± 6.8 |  |
| Involvements |  |  |  |  |
| Multifocal bones | Unifocal | 109 (37.1) | 61.4 ± 4.7 | 0.135 |
|  | Multifocal | 185 (62.9) | 51.1 ± 3.7 |  |
| Bone | No | 28 (9.5) | 50.0 ± 9.4 | 0.442 |
|  | Yes | 266 (90.5) | 55.5 ± 3.1 |  |
| Skin | No | 226 (76.9) | 61.7 ± 3.3 | < 0.001 |
|  | Yes | 68 (23.1) | 32.4 ± 5.7 |  |
| Liver | No | 258 (87.8) | 58.3 ± 3.1 | < 0.001 |
|  | Yes | 36 (12.2) | 30.6 ± 7.7 |  |
| Spleen | No | 273 (92.9) | 57.0 ± 3.0 | < 0.001 |
|  | Yes | 21 (7.1) | 28.6 ± 9.9 |  |
| Hematologic system | No | 276 (93.9) | 56.3 ± 3.0 | 0.002 |
|  | Yes | 18 (6.1) | 33.3 ± 11.1 |  |
| Lung | No | 253 (86.1) | 56.3 ± 3.1 | 0.048 |
|  | Yes | 41 (13.9) | 46.3 ± 7.8 |  |
| Lymph nodes | No | 268 (91.2) | 56.2± 3.0 | 0.108 |
|  | Yes | 26 (8.8) | 42.3 ± 9.7 |  |
| Pituitary | No | 278 (94.6) | 56.0 ± 3.0 | 0.069 |
|  | Yes | 16 (5.4) | 37.5 ± 12.1 |  |
| Thymus | No | 284 (96.6) | 55.8 ± 3.0 | 0.045 |
|  | Yes | 10 (3.4) | 30.0 ± 14.5 |  |
| Ear | No | 274 (93.2) | 56.4 ± 3.0 | 0.018 |
|  | Yes | 20 (6.8) | 35.0 ± 10.7 |  |
| Treatment response at week 6 | Responders | 149 (50.7) | 64.2 ± 3.9 | < 0.001 |
|  | Non-responders | 145 (49.3) | 45.4 ± 4.1 |  |
| *BRAF*-V600E mutation | Negative | 98 (33.3) | 56.8 ± 5.0 | 0.706 |
|  | Positive | 125 (42.5) | 52.7 ± 4.5 |  |
| Lymphocyte subsets^*^ |  |  |  |  |
| T cells (%) | < 55.3 | 90 (30.6) | 43.3 ± 5.2 | < 0.001 |
|  | ≥ 55.3 | 204 (69.4) | 60.1 ± 3.4 |  |
| B cells (%) | ≤ 22.8 | 179 (60.9) | 62.3 ± 3.6 | < 0.001 |
|  | > 22.8 | 115 (39.1) | 43.4 ± 4.6 |  |
| CD4^+^ T cells (%) | ≤ 58.7 | 207 (70.4) | 61.8 ± 3.4 | < 0.001 |
|  | > 58.7 | 87 (29.6) | 38.8 ± 5.3 |  |
| CD8^+^ T cells (%) | < 30.5 | 78 (26.5) | 39.6 ± 5.6 | < 0.001 |
|  | ≥ 30.5 | 216 (73.5) | 60.5 ± 3.3 |  |
| CD4/CD8 ratio | ≤ 1.6 | 164 (55.8) | 62.2 ± 3.8 | 0.004 |
|  | > 1.6 | 130 (44.2) | 45.7 ± 4.4 |  |
| Th1 cells (%) | < 9.3 | 162 (55.1) | 45.0 ± 3.9 | < 0.001 |
|  | ≥ 9.3 | 132 (44.9) | 67.1 ± 4.1 |  |
| Th1/Th2 ratio | < 12.2 | 89 (30.3) | 40.4 ± 5.2 | < 0.001 |
|  | ≥ 12.2 | 205 (69.7) | 61.3 ± 3.4 |  |
| Cytokines^*^ |  |  |  |  |
| IL-6 (pg/ml) | ≤ 10.5 | 102 (34.7) | 68.4 ± 4.6 | < 0.001 |
|  | > 10.5 | 192 (65.3) | 47.8 ± 3.6 |  |
| IL-10 (pg/ml) | ≤ 7.9 | 246 (83.7) | 59.9 ± 3.1 | 0.001 |
|  | > 7.9 | 48 (16.3) | 29.2 ± 6.6 |  |

^*^ Only the immune indicators with *P* values less than 0.05 in the results of ROC curve analysis were included in the univariate analysis.

**Supplementary Table S5** Receiver operating characteristic (ROC) curve analysis of lymphocyte subsets and cytokines in predicting prognosis of patients with MS-LCH.

| Indicators | AUC | 95% CI | *P*-value | Cut-off value |
| --- | --- | --- | --- | --- |
| Lymphocyte subsets (%) |  |  |  |  |
| T cells | 0.645 | 0.562-0.728 | 0.001 | 75.7 |
| B cells | 0.578 | 0.491-0.665 | 0.081 | 6.0 |
| NK cells | 0.55 | 0.464-0.636 | 0.260 | 4.3 |
| CD4^+^ T cells | 0.563 | 0.477-0.649 | 0.158 | 60.2 |
| CD8^+^ T cells | 0.556 | 0.47-0.642 | 0.206 | 30.5 |
| CD4/CD8 ratio | 0.532 | 0.446-0.619 | 0.467 | 2.1 |
| Th1 cells | 0.589 | 0.502-0.675 | 0.046 | 6.4 |
| Th2 cells | 0.504 | 0.417-0.592 | 0.923 | 0.7 |
| Th1/Th2 ratio | 0.574 | 0.489-0.66 | 0.095 | 10.9 |
| Th17 cells | 0.513 | 0.425-0.601 | 0.772 | 0.7 |
| Tregs | 0.518 | 0.431-0.606 | 0.681 | 6.6 |
| Treg/Th17 ratio | 0.5 | 0.411-0.589 | 0.999 | 12.5 |
| Cytokines (pg/ml) |  |  |  |  |
| IL-2 | 0.507 | 0.42-0.594 | 0.870 | 0.1 |
| IL-4 | 0.543 | 0.456-0.63 | 0.334 | 0.9 |
| IL-6 | 0.586 | 0.498-0.674 | 0.054 | 20.0 |
| IL-10 | 0.605 | 0.52-0.689 | 0.019 | 7.9 |
| TNF-α | 0.507 | 0.419-0.595 | 0.870 | 16.2 |
| IFN-γ | 0.583 | 0.498-0.668 | 0.061 | 1.3 |

**Supplementary Table S6** Univariate analysis of risk factors for progression-free survival (PFS) of MS-LCH.

| Variables | Groups | n (%) | 5-year PFS (% ± SE) | *P*-values |
| --- | --- | --- | --- | --- |
| Age | < 2 years | 81 (53.3) | 34.6 ± 5.3 | 0.004 |
|  | ≥ 2 years | 71 (46.7) | 57.3 ± 5.9 |  |
| Disease extent | MS RO^－^ LCH | 105 (69.1) | 51.2 ± 4.9 | 0.002 |
|  | MS RO^+^ LCH | 47 (30.9) | 31.9 ± 6.8 |  |
| Involvements | | | | |
| Multifocal bones | Unifocal | 48 (31.6) | 39.6 ± 7.1 | 0.245 |
|  | Multifocal | 104 (68.4) | 47.9 ± 4.9 |  |
| Bone | No | 22 (14.5) | 40.9 ± 10.5 | 0.661 |
|  | Yes | 130 (85.5) | 45.9 ± 4.4 |  |
| Skin | No | 87 (57.2) | 57.1 ± 5.4 | < 0.001 |
|  | Yes | 65 (42.8) | 29.2 ± 5.6 |  |
| Liver | No | 116 (76.3) | 49.8 ± 4.7 | 0.002 |
|  | Yes | 36 (23.7) | 30.6 ± 7.7 |  |
| Spleen | No | 131 (86.2) | 47.9 ± 4.4 | 0.038 |
|  | Yes | 21 (13.8) | 28.6 ± 9.9 |  |
| Hematologic system | No | 134 (88.2) | 46.8 ± 4.3 | 0.048 |
|  | Yes | 18 (11.8) | 33.3 ± 11.1 |  |
| Lung | No | 113 (74.3) | 44.9 ± 4.7 | 0.701 |
|  | Yes | 39 (25.7) | 46.2 ± 8.0 |  |
| Lymph nodes | No | 127 (83.6) | 46.3 ± 4.4 | 0.595 |
|  | Yes | 25 (16.4) | 40.0 ± 9.8 |  |
| Pituitary | No | 136 (89.5) | 46.1 ± 4.3 | 0.454 |
|  | Yes | 16 (10.5) | 37.5 ± 12.1 |  |
| Thymus | No | 142 (93.4) | 46.3 ± 4.2 | 0.292 |
|  | Yes | 10 (6.6) | 30.0 ± 14.5 |  |
| Ear | No | 133 (87.5) | 46.4 ± 4.3 | 0.408 |
|  | Yes | 19 (12.5) | 36.8 ± 11.1 |  |
| Treatment response at week 6 | Responders | 90 (59.2) | 59.7 ± 5.2 | < 0.001 |
|  | Non-responders | 62 (40.8) | 24.2 ± 5.4 |  |
| *BRAF*-V600E mutation | Negative | 38 (25) | 57.3 ± 8.1 | 0.183 |
|  | Positive | 71 (46.7) | 40.8 ± 5.8 |  |
| Lymphocyte subsets and cytokines ^*^ | | | | |
| T cells (%) | < 75.7 | 61 (40.1) | 45.7 ± 4.1 | 0.823 |
|  | ≥ 75.7 | 91 (59.9) | 33.2 ± 19.2 |  |
| Th1 cells (%) | ≤ 6.4 | 102 (67.1) | 34.3 ± 5.8 | 0.019 |
|  | > 6.4 | 50 (32.9) | 53.8 ± 5.8 |  |
| IL-10 (pg/ml) | ≤ 7.9 | 117 (77) | 53.6 ± 4.6 | < 0.001 |
|  | > 7.9 | 35 (23) | 17.1 ± 6.4 |  |

^*^ Only the immune indicators with *P* values less than 0.05 in the results of ROC curve analysis were included in the univariate analysis.

**Supplementary Table S7** Receiver operating characteristic curve analysis of lymphocyte subsets and cytokines in predicting prognosis of patients with SS-LCH

| Indicators | AUC | 95% CI | *P*-value | Cut-off value |
| --- | --- | --- | --- | --- |
| Lymphocyte subsets (%) |  |  |  |  |
| T cells | 0.584 | 0.492-0.676 | 0.086 | 59.0 |
| B cells | 0.523 | 0.425-0.62 | 0.641 | 17.2 |
| NK cells | 0.57 | 0.474-0.665 | 0.152 | 13.1 |
| CD4^+^ T cells | 0.575 | 0.48-0.671 | 0.122 | 51.2 |
| CD8^+^ T cells | 0.545 | 0.453-0.637 | 0.355 | 49.1 |
| CD4/CD8 ratio | 0.553 | 0.458-0.647 | 0.281 | 1.5 |
| Th1 cells | 0.559 | 0.466-0.653 | 0.223 | 9.5 |
| Th2 cells | 0.521 | 0.429-0.613 | 0.668 | 0.3 |
| Th1/Th2 ratio | 0.543 | 0.45-0.636 | 0.380 | 22.6 |
| Th17 cells | 0.526 | 0.433-0.619 | 0.600 | 0.9 |
| Tregs | 0.53 | 0.431-0.628 | 0.545 | 7.2 |
| Treg/Th17 ratio | 0.502 | 0.408-0.597 | 0.963 | 10.8 |
| Cytokines (pg/ml) |  |  |  |  |
| IL-2 | 0.531 | 0.436-0.626 | 0.528 | 0.1 |
| IL-4 | 0.528 | 0.434-0.622 | 0.566 | 1.0 |
| IL-6 | 0.558 | 0.468-0.647 | 0.236 | 3.3 |
| IL-10 | 0.501 | 0.405-0.597 | 0.976 | 3.1 |
| TNF-α | 0.523 | 0.426-0.619 | 0.641 | 3.6 |
| IFN-γ | 0.505 | 0.409-0.601 | 0.918 | 0.2 |


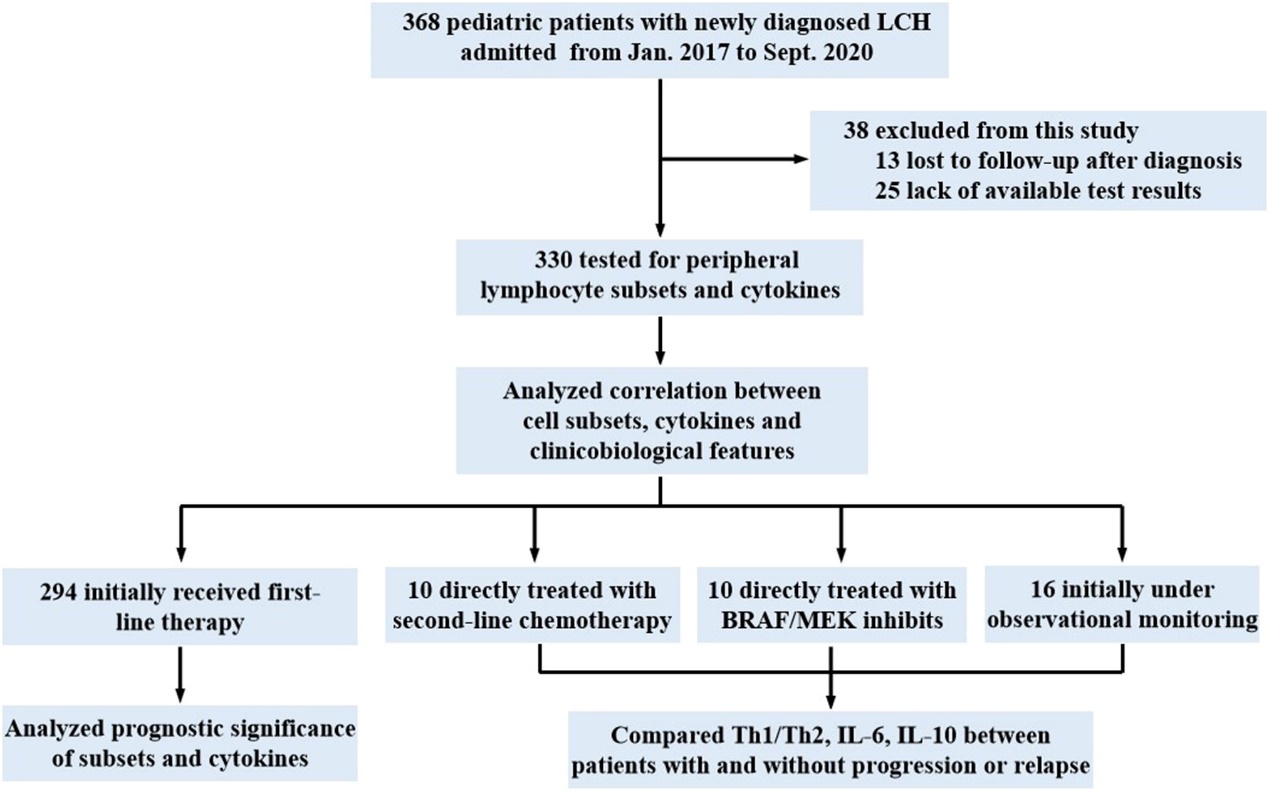


**Supplementary Figure S1** Flowchart of patient enrollment.

**
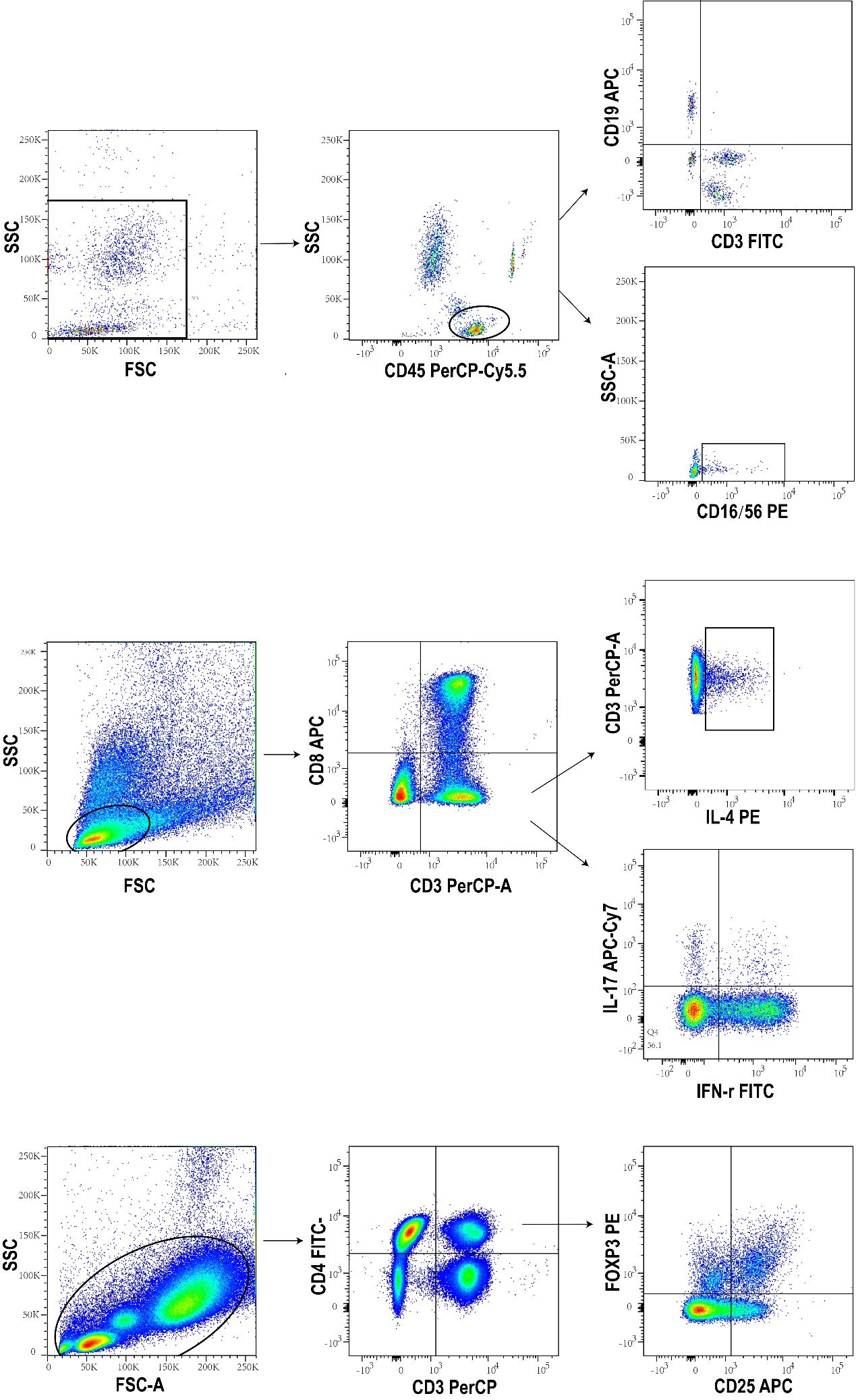
**

**Supplementary Figure S2** Representative flow cytometry gating strategy for Peripheral lymphocyte subsets in pediatric Langerhans cell histiocytosis.


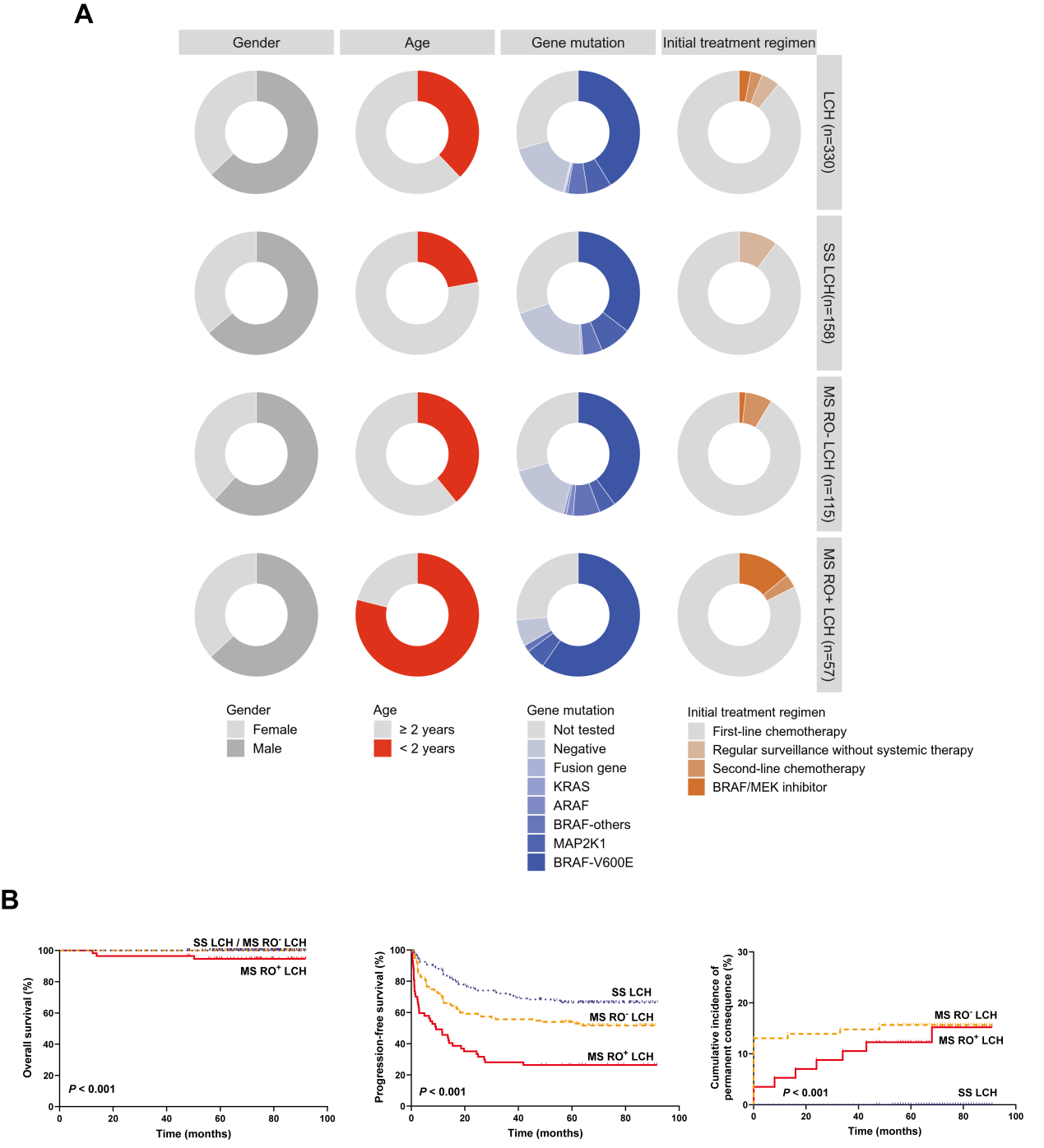


**Supplementary Figure S3** Baseline characteristics and prognosis of the enrolled patients. (A) Donut plots illustrating the clinicobiological features and initial treatment protocols for the entire cohort, as well as the three subgroups with different disease extents. (B) Kaplan-Meier curves representing overall survival, progression-free survival and cumulative incidence of permanent consequences for patients across the three clinical subgroups.


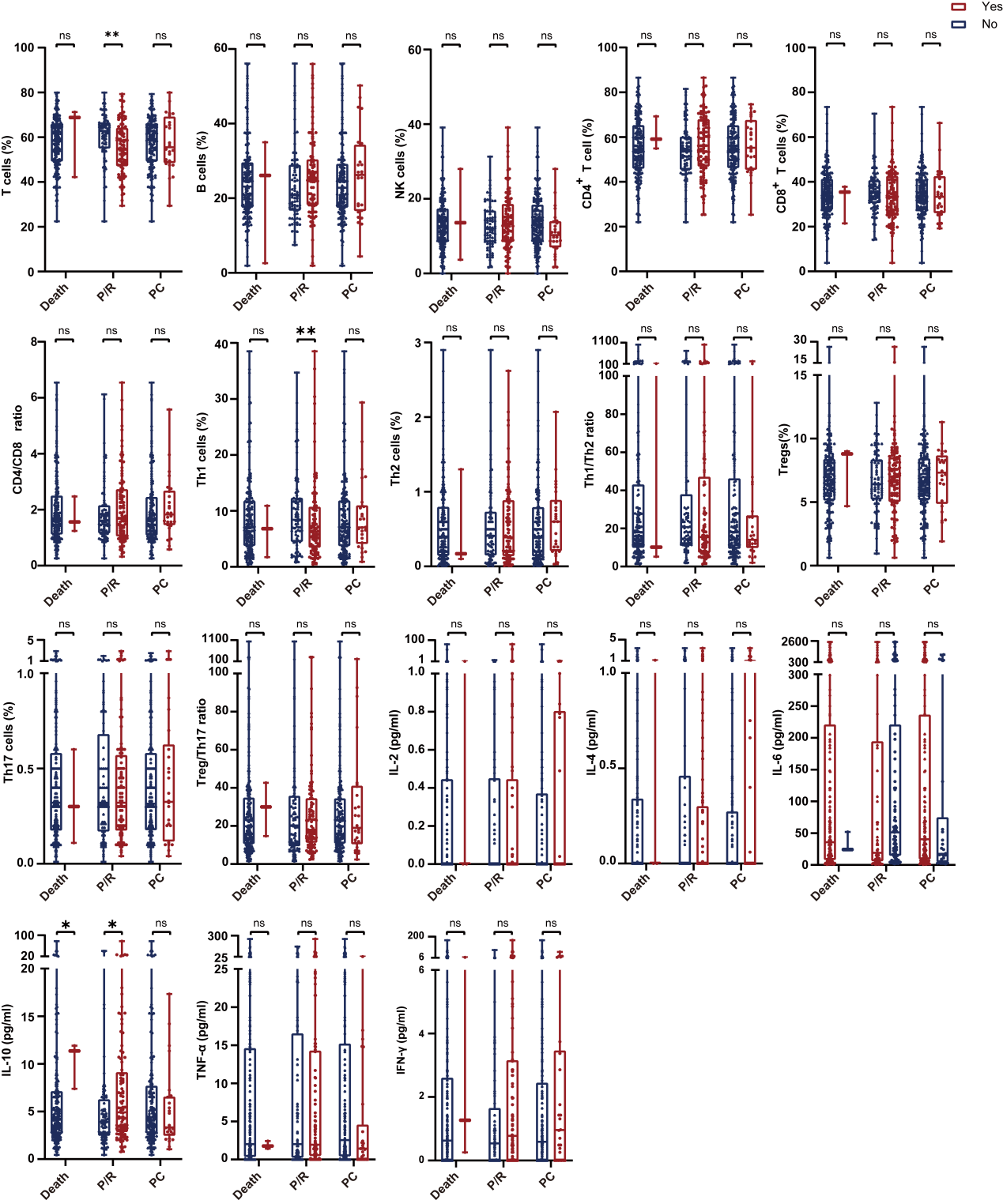


**Supplementary Figure S4** Box and whisker plots comparing lymphocyte subset proportions and cytokine levels between MS-LCH with or without events. Abbreviations: P/R, progression/relapse; PC; permanent consequences.


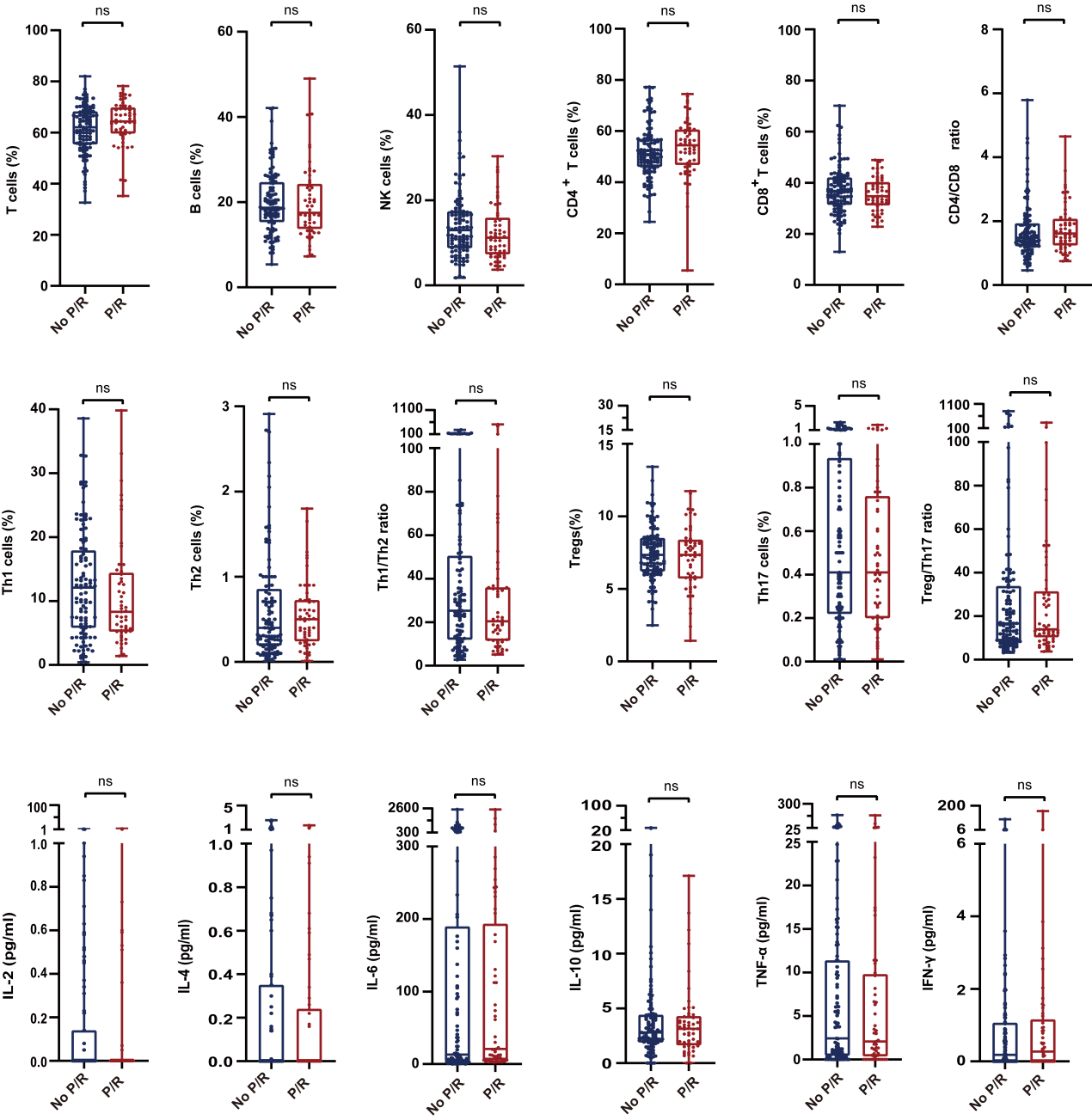


**Supplementary Figure S5** Box and whisker plots comparing lymphocyte subset proportions and cytokine levels between SS-LCH with or without progression/relapse. No comparative analysis could be conducted between these two patient groups due to the absence of mortality cases or sequelae in the SS-LCH cohort. Abbreviations: P/R, progression/relapse.


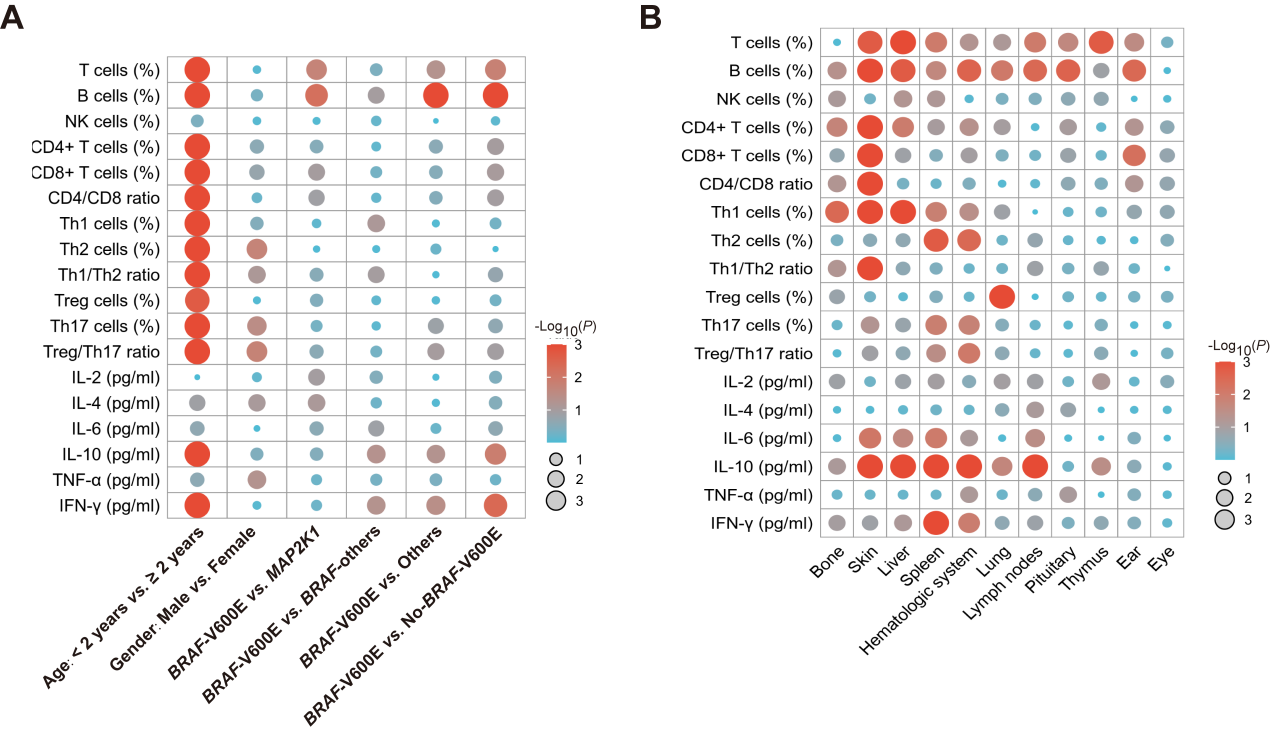


**Supplementary Figure S6** Bubble plot comparing lymphocyte subset proportions and cytokine levels in pediatric LCH patients with distinct clinical-biological features. (A) Comparison with different age, gender, or genetic mutations. (B) Comparison with different organ/system involvement. Each column represents a comparison between involved and non-involved patients.


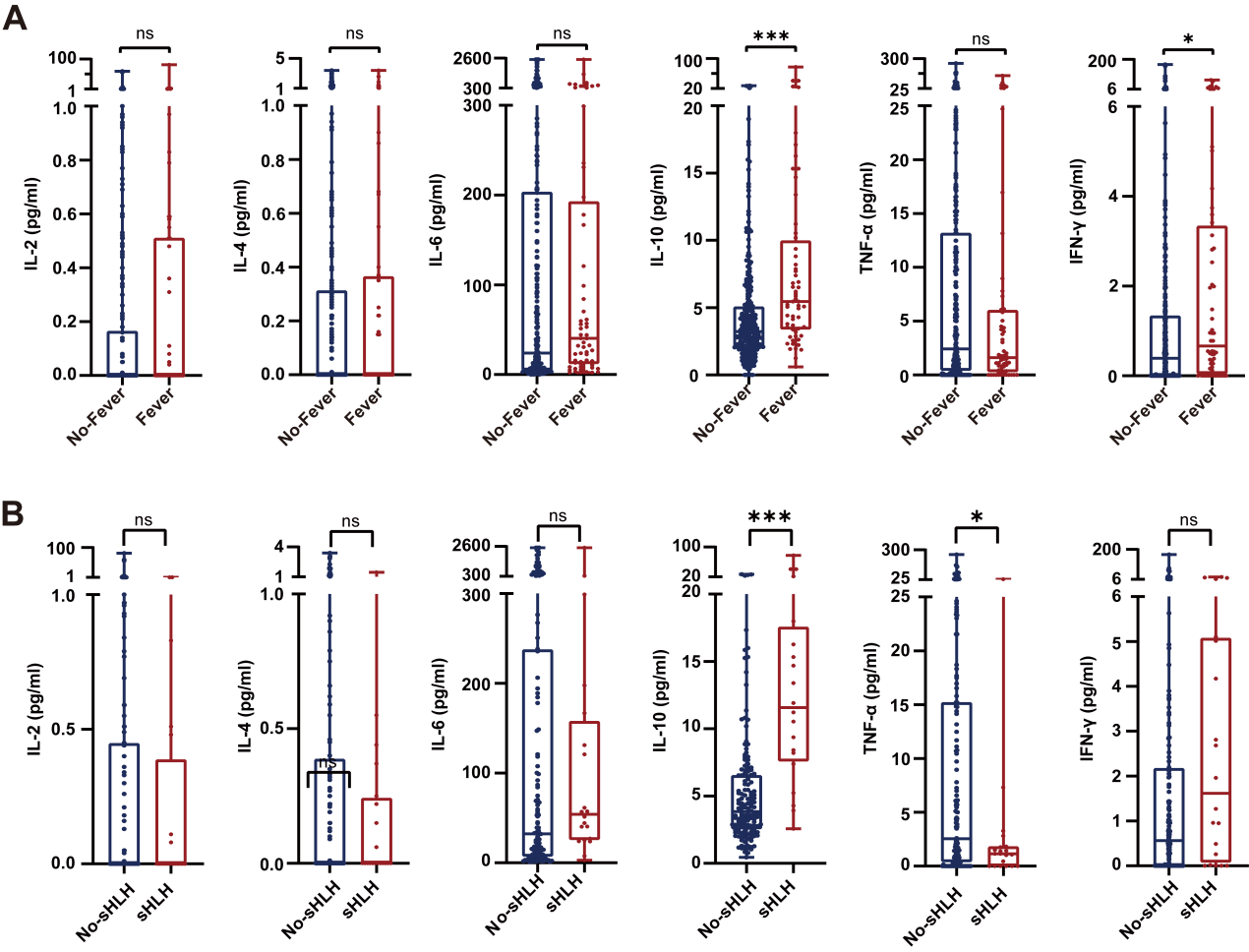


**Supplementary Figure S7** Box and whisker plots illustrating the relationship between fever, secondary hemophagocytic lymphohistiocytosis (HLH), and the proportions of subsets and cytokine levels: (A) Comparison of differences between patients with and without fever at diagnosis. (B) Differences between MS LCH patients with and without sHLH.


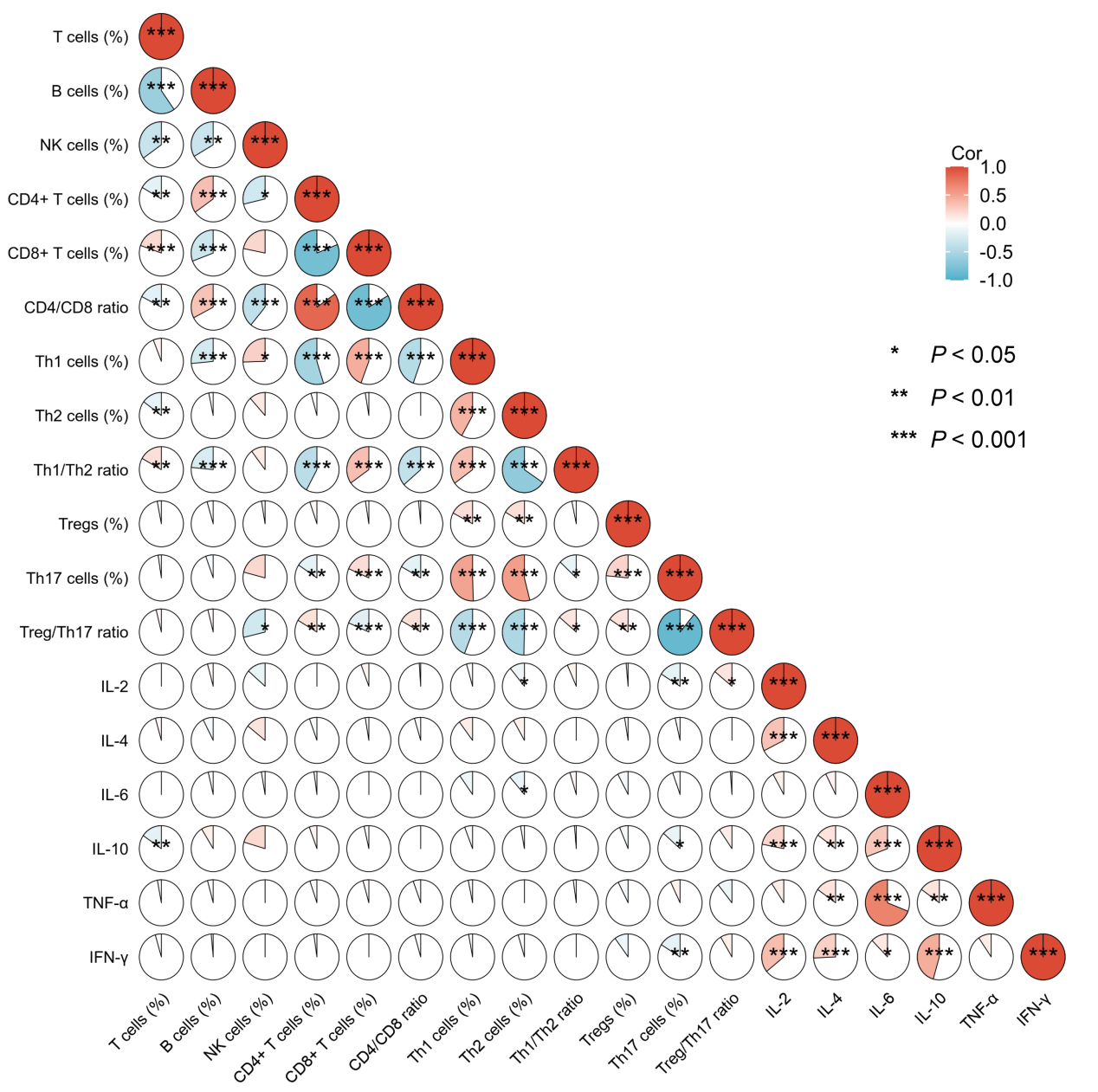


**Supplementary Figure S8** Pie plot showing the correlations among the lymphocyte subsets and cytokines.


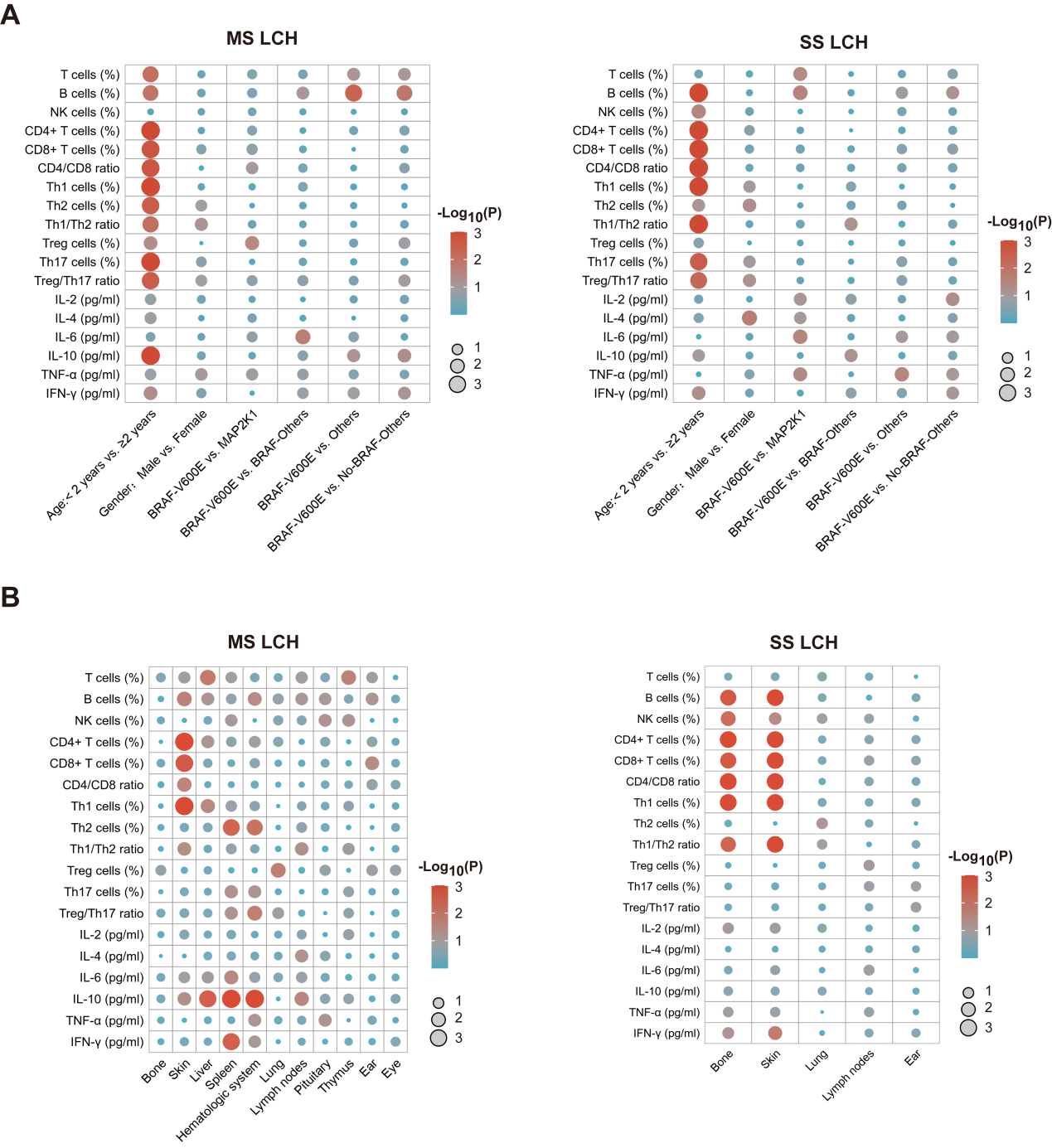


**Supplementary Figure S9** Bubble plot comparing lymphocyte subset proportions and cytokine levels in MS-LCH and SS-LCH patients with distinct clinical-biological features. (A) Comparison with different age, gender, or genetic mutations. (B) Comparison with different organ/system involvement.


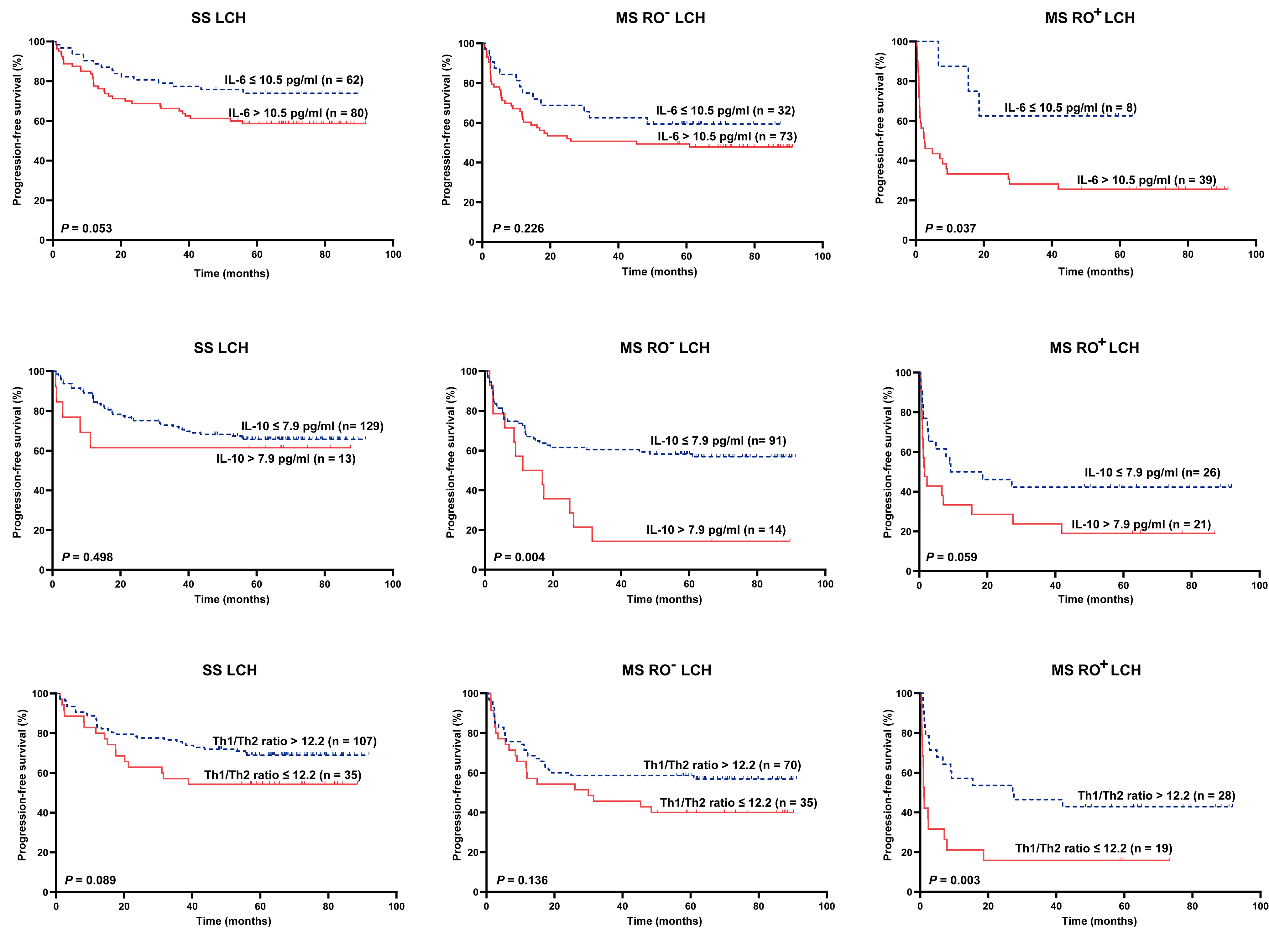


**Supplementary Figure S10** Kaplan-Meier survival curves according to IL-6, IL-10 and Th1/Th2 ratio in LCH patients at different disease extents.


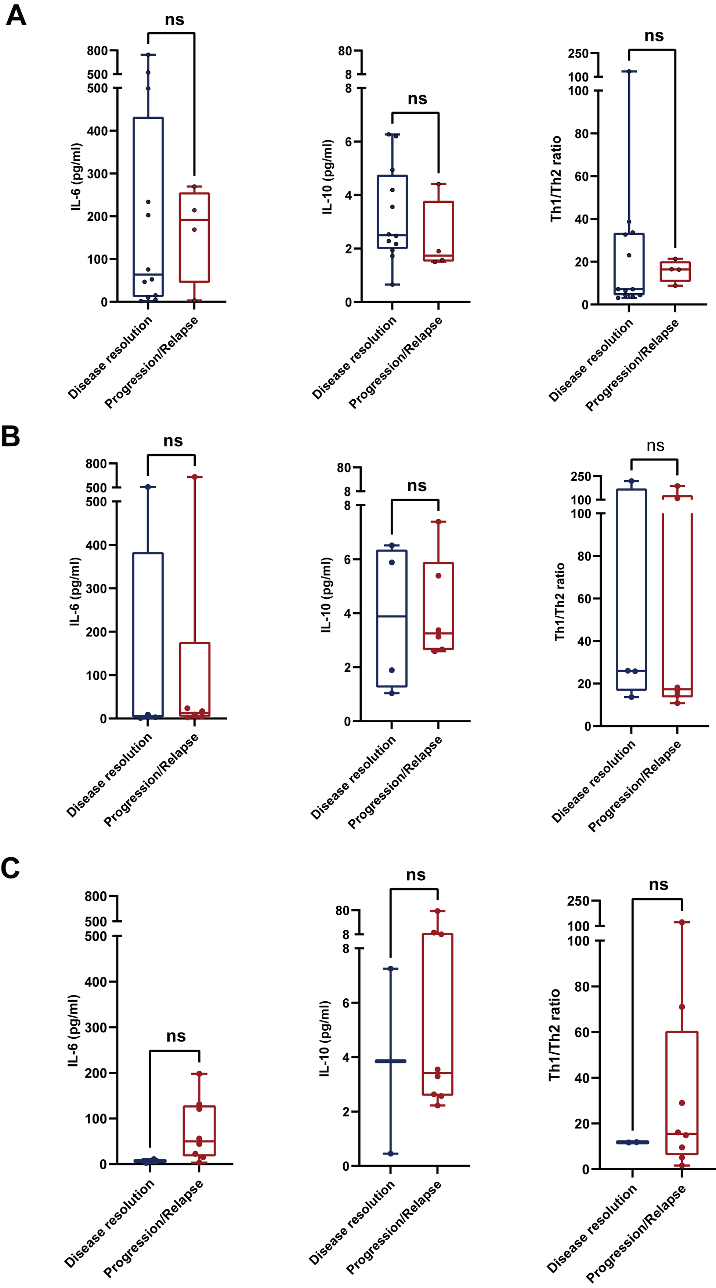


**Supplementary Figure S11** Boxplot graph showing the comparison of IL-6, IL-10 and Th1/Th2 ratio in patients treated with different treatment. (A) In the patients initially observed and monitored. (B) In the patients initially treated with second-line chemotherapy. (C) In the patients initially treated with targeted therapy.


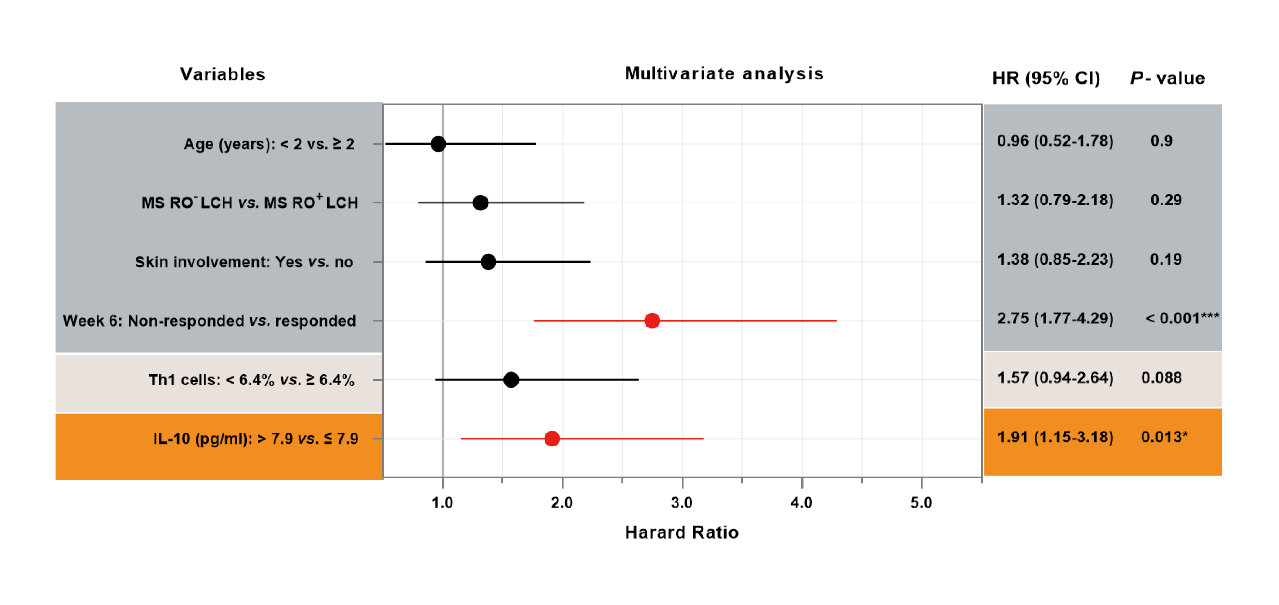


**Supplementary Figure S12** Forest plot of multivariate Cox regression analysis of risk factors for PFS of MS-LCH patients.


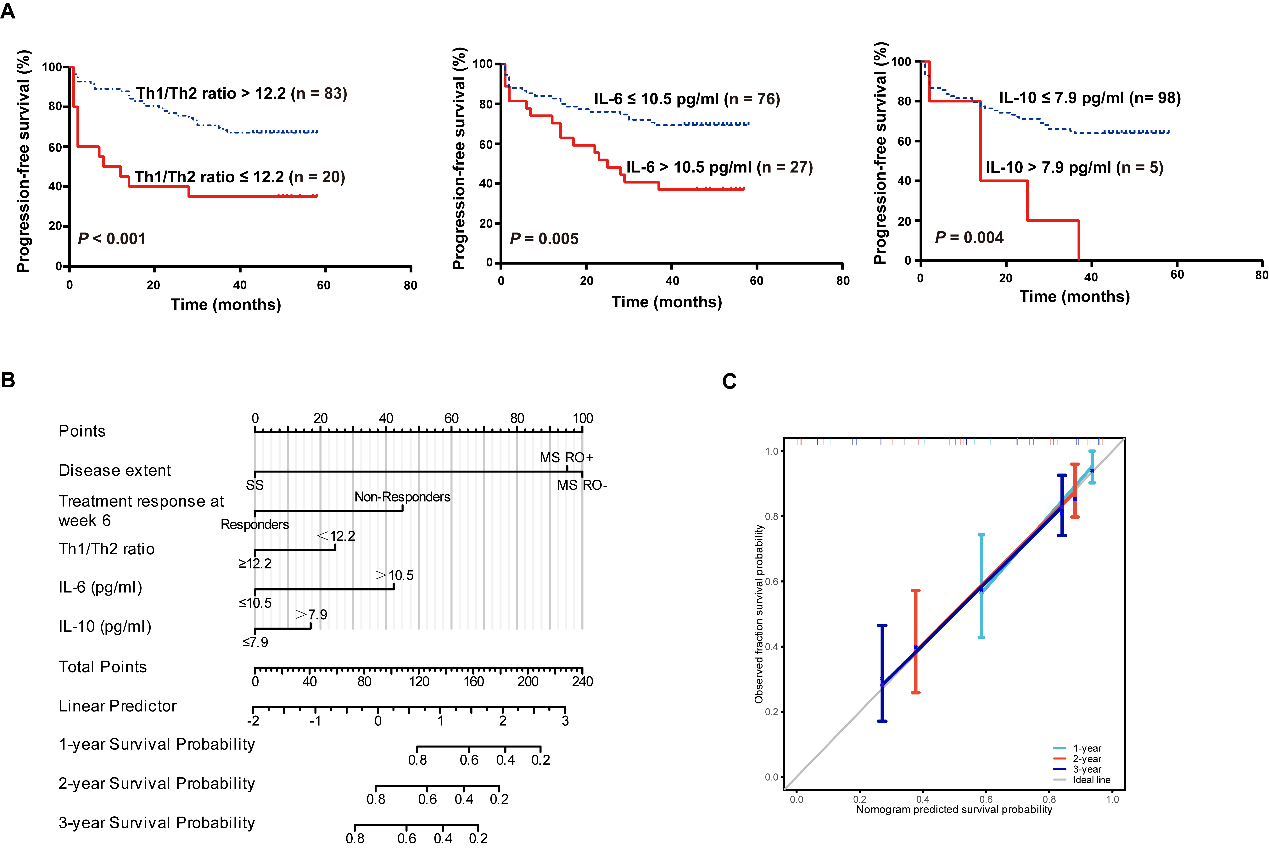


**Supplementary Figure S13** External Validation in 103 pediatric LCH patients. (A) Kaplan-Meier survival curves using discovery cut-offs for Th1/Th2, IL-6, and IL-10. (B) A nomogram model for predicting PFS. (C) Calibration plot of the nomogram for predicting PFS at 1-, 2-, and 3-years.
